# Comparison of VerifyNow-P2Y_12 _test and Flow Cytometry for monitoring individual platelet response to clopidogrel. What is the cut-off value for identifying patients who are low responders to clopidogrel therapy?

**DOI:** 10.1186/1477-9560-7-4

**Published:** 2009-05-06

**Authors:** Cosmo Godino, Loredana Mendolicchio, Filippo Figini, Azeem Latib, Andrew SP Sharp, John Cosgrave, Giliola Calori, Michela Cera, Alaide Chieffo, Alfredo Castelli, Attilio Maseri, Zaverio M Ruggeri, Antonio Colombo

**Affiliations:** 1 Department of Cardio-Thoracic and Vascular Diseases, San Raffaele Scientific Institute, Milan, Italy; 2 GVM Emo Centro Cuore Columbus, Milan, Italy; 3 Epidemiology and Medical Statistics Unit, San Raffaele Scientific Institute, Milan, Italy; 4 The Scripps Research Institute, Department of Molecular and Experimental Medicine, La Jolla, California, USA

## Abstract

**Background:**

Dual anti-platelet therapy with aspirin and a thienopyridine (DAT) is used to prevent stent thrombosis after percutaneous coronary intervention (PCI). Low response to clopidogrel therapy (LR) occurs, but laboratory tests have a controversial role in the identification of this condition.

**Methods:**

We studied LR in patients with stable angina undergoing elective PCI, all on DAT for at least 7 days, by comparing: 1) Flow cytometry (FC) to measure platelet membrane expression of P-selectin (CD62P) and PAC-1 binding following double stimulation with ADP and collagen type I either in the presence of prostaglandin (PG) E_1_; 2) VerifyNow-P2Y_12 _test, in which results are reported as absolute P2Y_12_-Reaction-Units (PRU) or % of inhibition (% inhibition).

**Results:**

Thirty controls and 52 patients were analyzed. The median percentage of platelets exhibiting CD62P expression and PAC-1 binding by FC evaluation after stimulation in the presence of PG E_1 _was 25.4% (IQR: 21.4–33.1%) and 3.5% (1.7–9.4%), respectively. Only 6 patients receiving DAT (11.5%) had both values above the 1^st ^quartile of controls, and were defined as LR. Evaluation of the same patients with the VerifyNow-P2Y_12 _test revealed that the area under the receiver-operating-characteristic (ROC) curve was 0.94 (95% CI: 0.84–0.98, p < 0.0001) for % inhibition and 0.85 (0.72–0.93, p < 0.005) for PRU. Cut-off values of ≤ 15% inhibition or > 213 PRU gave the maximum accuracy for the detection of patients defined as having LR by FC.

**Conclusion:**

In conclusion our findings show that a cut-off value of ≤ 15% inhibition or > 213 PRU in the VerifyNow-P2Y_12 _test may provide the best accuracy for the identification of patients with LR.

## Background

Thienopyridines such as clopidogrel inhibit P2Y_12_, one of two platelet adenosine diphosphate (ADP) receptors (P2Y_1_, P2Y_12_) and have been shown to confer clinical benefit in a variety of conditions characterized by the risk of arterial thrombosis [1-3]. Nonetheless, in the setting of coronary artery disease, about 1–1.9% of patients may experience acute or sub-acute stent thrombosis (ST) after implantation of a coronary stent [4,5] despite treatment with clopidogrel in combination with aspirin. Several mechanisms could explain a low platelet response to clopidogrel, including poor compliance to treatment, [6] variable absorption of the drug and/or variable generation of the active metabolite, and potential drug-drug interactions [7]. Some studies contend that resistance to clopidogrel may be present in as many as 20% of subjects [8-10]. Patients with a low response to clopidogrel are known to have an increased risk of cardiovascular events [11,12]. Thus, there is a clinical need for a reliable test of platelet response to clopidogrel therapy as a guide to individualizing dosing regimens. However, the ideal method for quantifying inhibition of platelet function by clopidogrel has yet to be agreed upon by the European Society of Cardiology and American College of Cardiology. Current assays which might be considered to be the gold standard, such as light transmission aggregometry (LTA), flow cytometric evaluation of platelet activation markers and flow cytometric measurement of the vasodilator-stimulated phosphoprotein (VASP) phosphorylation status, are technically complex and restricted to specialized laboratories and therefore none stands out as the clear investigation of choice.

On the other hand, the accuracy of point-of-care assays is still unclear [13,14]. The VerifyNow-P2Y_12 _test was designed to overcome the limitations of conventional optical platelet aggregation assays. It is a rapid test that uses ADP to stimulate platelets in the presence of prostaglandin (PG) E_1_, which inhibits activation downstream of a second ADP receptor P2Y_1_, thus making the assay more sensitive to the activity of P2Y_12_. The test can be performed directly in the catheterization laboratory prior to percutaneous coronary intervention (PCI). However, there are minimal clinical data on which to define a suitable cut-off value for low responders [15-17]. The aim of our study was to compare the VerifyNow-P2Y_12 _test with flow cytometric analysis of platelet activation to define a cut-off value for low responders to clopidogrel.

## Methods

### Study population

After obtaining institutional approval and informed consent, we studied 52 consecutive patients aged 64 ± 11 years (mean ± SD) who had evidence of stable coronary artery disease and were undergoing elective PCI. All patients studied were on dual anti-platelet therapy (DAT), 100 mg aspirin and 75 mg clopidogrel daily, for at least 7 days prior to testing. Patients who had received intravenous heparin, abciximab, tirofiban, or eptifibatide in the previous week (typically, patients with acute myocardial infarction or refractory unstable angina) were excluded from the study, as were patients with a known platelet function disorder or a preoperative hematocrit or platelet count outside the ranges validated for the VerifyNow-P2Y_12 _test (33–52% and 119.000–502.000/μL, respectively).

Thirty medication-free volunteers aged 45 ± 16 years, who were apparently healthy and had no known risk factors for coronary artery disease, were enrolled from within the medical staff of our hospital and acted as controls. All studies involving human subjects were conducted in accordance with the Declaration of Helsinki.

### Platelet Function Analysis

The response to clopidogrel was assessed by flow cytometric analysis of platelet activation, considered as the reference standard in this study, and by the VerifyNow-P2Y_12 _test. Blood specimens were obtained from an antecubital vein through a clean venipuncture with minimal stasis using a 19-gauge needle and the double syringe method, in which the first five mls of blood were discarded to avoid spontaneous platelet activation [18]. Samples were processed within 1 hour after venipuncture by two different groups of operators. For the VerifyNow-P2Y_12 _test, blood was transferred into two 1.8-ml blood collection tubes containing 0.2 ml buffered 3.2% Na_3_-citrate solution (Venoject/Venosafe, Terumo Europe N.V., Leuven, Belgium) and analyzed after at least 10 minutes and within 2 hours as suggested by the manufactures.

#### Flow cytometry analysis of platelet activation

Platelet activation was assessed in platelet-rich plasma (PRP) as previously described [19] in accordance with the European Working Group consensus protocol for the flow cytometric characterization of platelet function. We chose PRP, rather than whole blood, in order to avoid the potential for release of ADP by red blood cells which might stimulate platelet activation. Two mAbs were used: fluorescein isothiocyanate (FITC)-conjugated PAC-1 (Beckton Dickinson, San Jose, CA), which binds to the activated conformation of GP IIb-IIIa, and an anti-P selectin mAb (also known as CD62P)labelled with R-phycoerythrin (PE) (Caltag Laboratories, Burlingame, CA). P-selectin is present on the surface of activated but not resting platelets. These two activation markers were analyzed on individual platelets in PRP visualized in a flow cytometer (FC500; Beckman Coulter, S.p.A., Cassina De' Pecchi, Milan, Italy) before and after double stimulation, for 110 min at room temperature (22–25°C), with 20 μM ADP (Sigma Chemical Co., St. Louis, MO) and 5 μg/mL equine tendon collagen type I (Nycomed Pharma GmbH, Germany) in presence of 1 μM PG E_1 _(Sigma) in order to prevent artifact caused by sample manipulation and to ensure sample stability over time. The ADP/collagen combination acts as a strong stimulus in unstirred PRP, equivalent to α-thrombin that cannot be used because it causes fibrinogen clotting. Forward light scatter (FS) and side light scatter (SS) were displayed on logarithmic scales, and the instrument settings were chosen to highlight the platelet population that represented >95% of the elements in PRP. Platelets were also positively identified by binding of an anti-GP Ibα (CD42b) mAb labeled with FITC (Immunotech, Marseille Cedex, France). Samples were analyzed with the acquisition of 20000 events. The fluorescence of each identified particle was represented on a log scale. All mAbs were used at saturating concentrations and were incubated for 15 minutes at room temperature after platelet stimulation or in unstimulated PRP. We did not use fixative solutions because all the ones we tested contributed to platelet activation and/or interfered with antibody binding. At the end of the incubation with antibodies, the samples were diluted with phosphate-buffered saline (PBS, 0.04 M phosphate buffer and 0.14 M NaCl, pH 7.4) and immediately analyzed in the flow cytometer. For all antibodies used, a control antibody of the same IgG isotype and labelled with the same fluorochrome was incubated with PRP under identical conditions, and platelets were then analyzed to set the lower limit of positive fluorescence. Results were expressed as percentage of positive platelets. The conditions described here are not standard reference conditions. Rather, these are standardized conditions chosen by our laboratory to ensure internal reproducibility.

#### VerifyNow-P2Y_12 _assay

The VerifyNow-P2Y_12 _test cartridge system (Accumetrics, San Diego, CA, USA; in Italy distributed by Endotech S.P.A., Como) was used as described previously [20]. This is a rapid (less than 5 minutes) platelet function assay designed to measure directly the effects of drugs on the P2Y_12 _receptor. The assay is a turbidimetric-based optical detection system that, like optical aggregometry, depends on the ability of activated platelets to bind fibrinogen. The assay contains 20 μmol ADP and 22 nmol PG E1 to reduce the activation contribution from ADP binding to P2Y_1 _receptors, thus making the assay specific for the effects of ADP mediated by P2Y_12_. The test is designed to measure platelet P2Y_12 _receptor blockade in patients already receiving clopidogrel, whose treatment cannot be discontinued solely to obtain a baseline level of platelet activity. Thus, the latter parameter (BASE) is determined using a modified thrombin receptor activating peptide (iso-TRAP) in a reference channel. The results of the assay (TEST) are reported as absolute P2Y_12_-Reaction-Units (PRU) as well as percent inhibition (% inhibition), the latter calculated as 100 - (TEST/BASE × 100) or [(BASE - TEST)/BASE] × 100.

### Evaluation of "Controls" and definition of "Low response" to clopidogrel at Flow Cytometry

In healthy volunteers, the median percentages of platelets stimulated in the presence of PG E_1 _that expressed CD62P was 25.4% (IQR: 21.4–33.1%). The corresponding values for PAC-1 binding were 3.5% (IQR: 1.7–9.4%). CD62P expression and PAC-1 binding showed no correlation with age (Spearman r = 0.17 and 0.16, respectively). The median percentages of platelets exhibiting CD62P expression and PAC-1 binding in controls aged >30 years (54 ± 12.4, mean ± SD) or <30 years (27 ± 2, mean ± SD) were 24% vs. 23.6% and 4.8% vs. 2.1%, respectively, and were not significantly different.

A patient was defined as a *"Low-responder" *to clopidogrel when the percentages of platelets exhibiting CD62P expression **and **PAC-1 binding were **both **above the 1^st ^quartile of normal distribution (21.4% and 1.7%, respectively); and as a *"High-responder" *when **both **values were below the 1^st ^quartile of normal. The remaining patients (with percentage of platelets expressing CD62P above the 1^st ^quartile of normal but percentage of PAC-1 binding platelets below the 1^st ^quartile of normal, or vice versa) were considered an "*Intermediate-responder" *to clopidogrel.

### Statistics

The results of the assays were not normally distributed, as verified using the Kolmogorov-Smirnov test. Thus they are reported here as median and interquartile range (IQR) unless otherwise stated. Differences between groups were analyzed with the Mann-Whitney test and Spearman or Pearson testing was appropriately performed for correlation analysis between variables. The diagnostic value of the VerifyNow-P2Y_12 _test was assessed by calculating the area under receiver-operating characteristic (ROC) curves which plot sensitivity (true-positive fraction) versus 1-specificity (where the latter is the false-positive fraction) for a range of results [21]. The values of PRU and % inhibition yielding the highest accuracy (maximal true positive and minimal false positive results, where positive was defined by the results of the flow cytometric assay) where chosen as cut-off limits of the VerifyNow-P2Y_12 _test. Statistical analyses were performed using SPSS 16 (SPSS, Inc., Chicago, Illinois), MEDCALC 9.3 (MedCalc Software, Mariakerke, Belgium) and GraphPad 5.00 (GraphPad Software, San Diego, California). All statistical tests were two-tailed and p values < 0.05 were considered significant.

## Results

### Flow Cytometry

Fifty-two patients (age, 64 ± 11 years, mean ± SD) were tested and their clinical characteristics are reported in Table 1. By flow cytometric analysis, the percentages of platelets that expressed CD62P or bound PAC-1 (median and IQR) were 11.9% (1.8–43%) and 1.8% (0.1–8%) respectively, both significantly lower (p < 0.0002 and p < 0.007, respectively) than the corresponding values in normal controls. Among the patients, six (11.5%) exhibited both CD62P expression and PAC-1 binding above the 1^st ^quartile of normal (median and IQR: 29.5%, 23.4–42.9%; and 4.4%, 2.3–7.9%, respectively). These six patients were therefore defined as low-responders to clopidogrel. In contrast, 23 patients (44.2%) exhibited both CD62P expression and PAC-1 binding below the 1^st ^quartile of normal (median and IQR: 7.8%, 1.8–19.7%; and 0.8%, 0.1–1.7%, respectively) and were defined as high-responders to clopidogrel. The remaining 23 patients (44.2%) were defined as intermediate responders, due to a high (above the 1^st ^quartile of normal) percentage of platelets expressing CD62P and a low (below the 1^st ^quartile of normal) percentage of platelets binding PAC-1 (2 patients, 3.8%) or vice versa (21 patients, 40.3%). There were no differences in baseline clinical characteristics between patients defined as low-responders to clopidogrel versus intermediate plus high-responder patients, (Table 2).

**Table 1:** Baseline Clinical Characteristics of 52 Patients.

	**N (%) or mean ± SD**
Age (years)	64 ± 11
Male	39 (75)
Ejection fraction %	53 ± 8
Body mass index >30, kg/m^2^	7 (15)
Hematocrit %	36.6 ± 8.3
Platelet count (× 10^3^/μL)	251 ± 127
**Risk Factors**	
Diabetes	14 (28)
-Insulin dependent Diabetes	6 (12)
Hypertension	32 (64)
Hypercholesterolemia	31 (62)
Current smoking	5 (10)
	
**History**	
Previous MI	23 (46)
Previous PCI	16 (48)
Previous CABG	6 (12)
Chronic renal failure†	2 (4)
***Coronary Artery Disease n, (%)***	
1 vessel	12 (28)
2 vessel	29 (55)
3 vessel	18 (34)
***Therapy n, (%)***	
Beta-blockers	32 (61)
ACE-inhibitors	30 (57)
Nitrates	10 (19)
Ca-antagonists	17 (32)
Statins	38 (71)

Values are expressed as means ± SDs or number (percentages) of patients.

MI = acute myocardial infarction; PCI = percutaneous coronary intervention; CABG = coronary artery bypass graft. †Defined as the presence of previously documented renal failure and/or a baseline serum creatinine level > 2 mg/dl.

**Table 2:** Correlation analysis of variables and clopidogrel low response.

**Variables**	Low Responder patients* N = 6	Intermediate plus High Responder patients* N = 46	**P value**
Age	62 ± 13.2	64 ± 10.7	0.671
Male	4 (66)	35 (76)	0.472
Body mass index, kg/m^2^	26 ± 3.3	26 ± 4.3	0.956
Ejection fraction %	54 ± 3.7	52 ± 9.6	0.652
Hematocrit %	36.2 ± 7.8	37 ± 8	0.774
Platelet count (× 10^3^/μL)	268 ± 124	235 ± 130	0.439
Current smoking	1 (17)	4 (87)	0.487
Diabetes	1 (17)	13 (29.5)	0.663
-Insulin dependent Diabetes	0	6 (15)	0.444
Chronic renal failure	0	2 (43)	0.772
Hypertension	3 (50)	29 (63)	0.369
Hypercholesterolemia	3 (50)	28 (61)	0.412
Statins	6 (100)	32 (70)	0.136
CYP3A4-metabolizing statins	5 (83)	23 (50)	0.134

Values are expressed as means ± SDs or number (percentages) of patients.

*Defined by flow cytometric evaluation of platelet activation parameters.

As expected, the median values of CD62P expression and PAC-1 binding in patients were significantly lower than in controls only in high responders (p < 0.0001) and intermediate responders (p < 0.0001 and p < 0.02, respectively).

### VerifyNow-P2Y_12 _test

**Figure 1:**
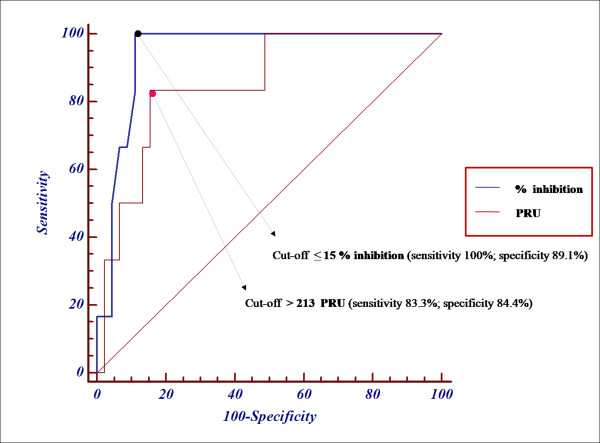
**Receiver Operator Characteristic (ROC) curves defining the sensitivity and specificity of the VerifyNow-P2Y_12 _test in patients receiving antiplatelet therapy**. Sensitivity and specificity in discriminating responders from non-responders to treatment was based on the arbitrary definition of positive patients (*Low responders *to clopidogrel; n = 6) and negative patients (*High *plus *Intermediate responders *to clopidogrel; n = 46) based on the percentage of platelets exhibiting CD62P expression and PAC-1 binding after stimulation in the presence of PG E_1_. The two curves shown here, for results expressed as PRU (absolute P2Y_12 _Reaction Unit) and % of inhibition (% inhibition), respectively, define cut-off values of PRU > 213 and ≤ 15% inhibition, as those giving the highest accuracy (minimal false negative and positive results) in the VerifyNow-P2Y_12 _test.

With the VerifyNow-P2Y_12 _test, the six patients defined as low-responders to clopidogrel by flow cytometric analysis of platelet activation gave a median % inhibition value of 9.5% (IQR: 9–11.5%). In contrast, the 23 patients defined as high-responders gave a median % inhibition value of 43% (IQR: 34–68%). The differences between these results was significant (p < 0.0007). The area under the ROC curve for the VerifyNow-P2Y_12 _test values expressed as % inhibition was 0.94 (95% confidence interval, 0.84–0.98; p < 0.0001; Figure 1). From the ROC curve analysis, a cut-off of ≤ 15% inhibition gave the maximum accuracy (sensitivity 100%; specificity 89.1%). Considering the same results expressed as absolute PRU, the area under the ROC curve was 0.85 (95% confidence interval, 0.72–0.93; p < 0.005; Figure 1) with a cut-off for accuracy of > 213 (sensitivity 83.3%; specificity 84.4%).

**Figure 2:**
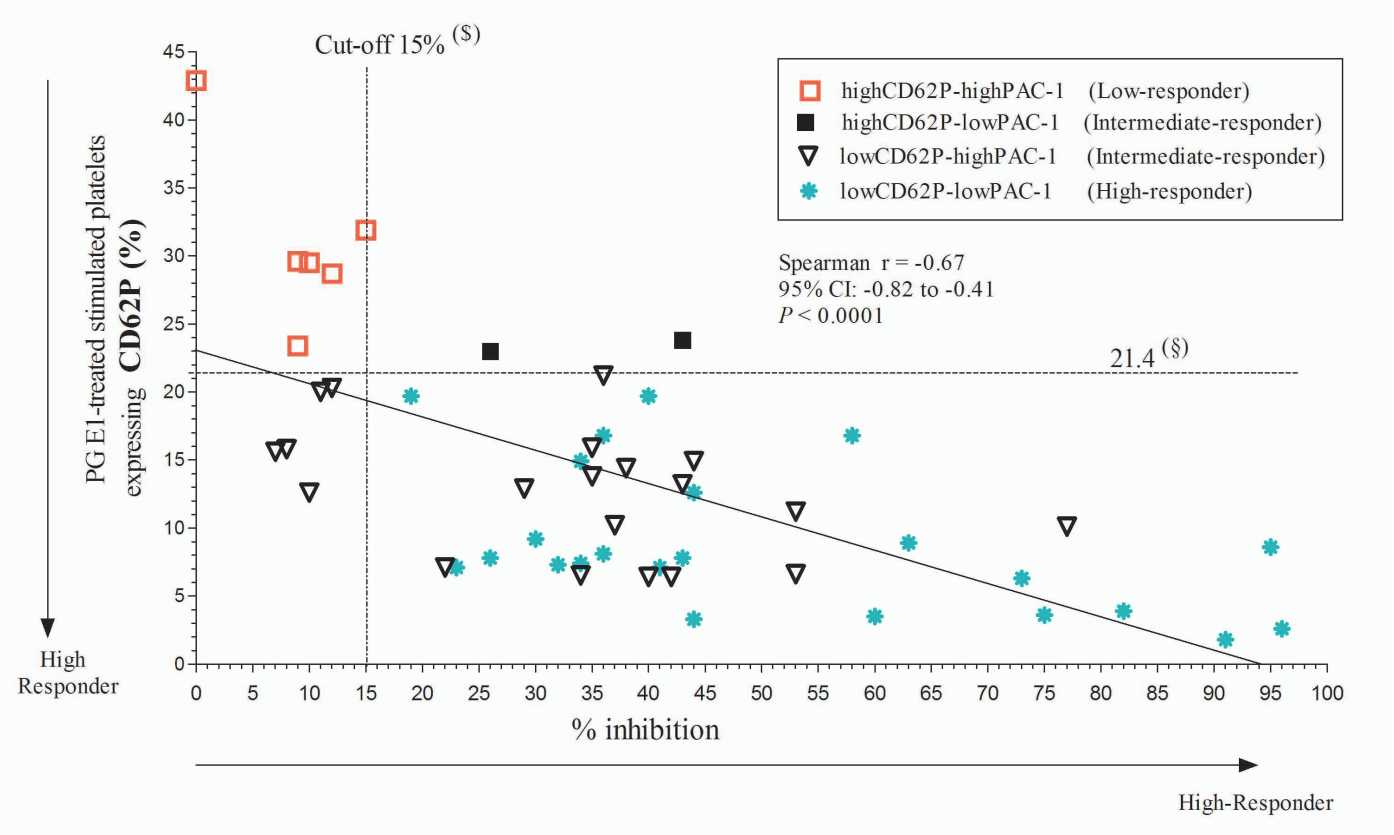
**Correlation between the results obtained by flow cytometric evaluation of platelet activation parameters and VerifyNow-P2Y_12 _test in patients receiving antiplatelet therapy**. The percent of inhibition (% inhibition) was plotted against the percentage of platelets that expressed P-selectin (CD62P) after stimulation with 20 μM ADP and 5 μg/mL collagen type I for 110 min at room temperature in the presence of 1 μM PG E_1_. (§) First quartile of control values for CD62P expression. Patients receiving antiplatelet therapy with levels of CD62P expression and PAC-1 binding above this limit were defined as "*Low-responder*"; those with both values below this limit were defined as "*High-Responder*"; those with one value above and the other below the corresponding limit were defined as "*Intermediate-responder*". ($) Cut-off value for % of inhibition in the VerifyNow-P2Y_12 _test giving minimal false negative and positive results (see Figure 1).

**Figure 3:**
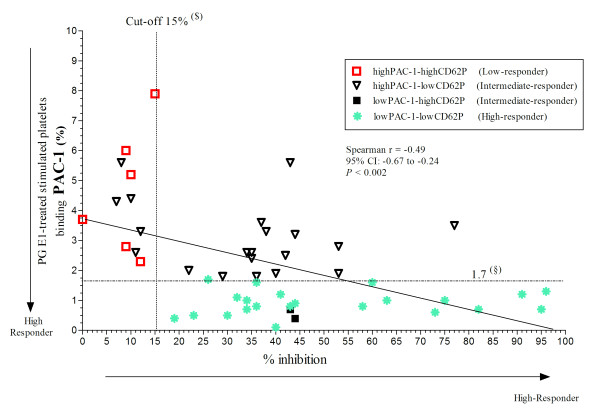
**Correlation between the results obtained by flow cytometric evaluation of platelet activation parameters and VerifyNow-P2Y_12 _test in patients receiving antiplatelet therapy**. The percent of inhibition (% inhibition) was plotted against the percentage of platelets that expressed bound PAC-1 after stimulation with 20 μM ADP and 5 μg/mL collagen type I for 110 min at room temperature in the presence of 1 μM PG E_1_. (§) First quartile of control values for PAC-1 binding. The others abbreviations as reported in the Figure 2.

All six patients (11.5%) defined as low-responders on the basis of CD62P expression and PAC-1 binding were lower or equal to 15% inhibition, and all 23 patients (44.2%) defined as high-responders were above 15% inhibition (Figure 2 and 3). Of the remaining 23 (44.2%) patients defined as intermediate responders, five were below 15% inhibition; in all these individuals, the percentage of platelets expressing CD62P was below or equal the 1^st ^percentile of normal controls (Figure 2), but that of platelets binding PAC-1 was above (Figure 3). In total 11 patients (21%) were lower or equal to 15% inhibition and as a result low clopidogrel responders.

Finally, a good correlation was found between % inhibition and the percentages of platelets that expressed CD62P or bound PAC-1 (Spearman r = -0.67, p < 0.0001; and -0.49, p < 0.002, respectively; Figure 2).

## Discussion

A simple test such as the VerifyNow-P2Y_12 _test may be useful to monitor the response to antiplatelet therapy and offers the possibility of a point-of-care approach, e.g. to assess whether the response to a clopidogrel loading dose is adequate in patients undergoing coronary stenting. As relevant as the simplicity of its use is, the correct interpretation of results remains of paramount importance to make appropriate therapeutic decisions. According to the manufacturer, the initial proposed cut-off value to discriminate patients with a low response to clopidogrel using the VerifyNow-P2Y_12 _test was 50% inhibition (not specified in the user manual, only suggested informally). Using such a value, we were surprised to find that an unexpectedly high 75% of our 52 patients treated with DAT were defined as low clopidogrel responders. We considered this as unlikely and sought to verify the validity of the proposed cut-off limit. Indeed, by using markers of platelet activation measured by flow cytometry to define low responders, we found by ROC curve evaluation that a % inhibition ≤ 15 and absolute PRU > 213 may be VerifyNow-P2Y_12 _cut-off values that identify patients with low response to clopidogrel with maximum sensitivity and specificity. Using the ≤ 15% inhibition limit that we established, only 21% of our 52 patients receiving conventional clopidogrel treatment were classified as low responders (and 23% using the PRU > 213 limit). This result is in agreement with other studies that have shown resistance to clopidogrel may be present in as many as 20% of subjects [8,10,22]. Recently, Lordkipanidzé et al. [23] suggested that the assay-reported % inhibition of platelets fails to accurately quantify inhibition achieved by clopidogrel, as TRAP-induced aggregation may not adequately mimic off-drug ADP/PGE1-induced aggregation. We have therefore described the usefulness of both the PRU value and the % inhibition value for detecting 'low responders' in this manuscript.

In the present study, the reliability of both cut-off values was shown to be high on ROC curve analysis, with the value for % inhibition shown to have a 100% sensitivity for detecting low responders as defined by flow cytometry, a finding that contrasts with the results obtained in the work by Lordkipanidzé et al. [23]. This emphasises the importance of further published studies in this area, as establishing the reliability of each assay may not be as straight forward as first appears. Whilst the accuracy of the % inhibition value quoted by the VerifyNow-P2Y12 test remains to be clarified, there are now several clinical studies which show that a high PRU obtained with this assay is associated with adverse clinical consequences. For example, in a large clinical study of 380 PCI patients, a high PRU was strongly associated with adverse clinical outcomes, including stent thrombosis (p = 0.004) and furthermore was associated with the hardest of clinical endpoints – cardiovascular death (p = 0.04) [15]. Subsequent publications have confirmed the usefulness of the VerifyNow assay in predicting adverse clinical events [16,17]. The usefulness of our lab cut-off value of PRU > 213 should be considered in conjunction with other published cut-offs proven be of clinical significance, such as those identified by Price et al. (PRU > 235) [15] and Patti et al. (PRU > 240) [16]. From these data, we can infer that whilst the % inhibition value remains of interest, the PRU value (a direct measure of platelet aggregation induced by either ADP/PGE1) may be a more reliable method of identifying subjects who are hyporesponsive to clopidogrel therapy and subsequently at risk of clinical events.

The validity of our conclusions is entirely dependent on the definition of resistance to anti-platelet therapy, for which we relied on the measurement of two platelet activation markers by flow cytometric analysis. Flow cytometry, similarly to light transmission aggregometry (LTA), is used by specialized laboratories and represents a reference standard for the assessment of platelet function [13,14].

In this regard, LTA is the most widely used method but has well-known limitations, including poor reproducibility, high sample volume, requirement for sample preparation, length of assay time, requirement for a skilled technician, and cost [13]. Therefore, new options for platelet function testing have been developed to address these disadvantages and to meet the need for point-of-care testing that can be performed at or near a patient's bedside without requiring a high degree of technical expertise. The new tests include VerifyNow (Accumetrics, San Diego, CA); Plateletworks (Helena Laboratories, Beaumont, TX); Thrombelastograph PlateletMapping System (Haemoscope Corporation, Niles, IL); Impact cone and plate(let) analyzer (DiaMed, Cressier, Switzerland); and Platelet Function Analyzer 100 (PFA-100; Dade Behring, Newark, DE) [13]. However, the relationship of in vivo platelet function and adverse clinical events to results of ex vivo platelet function tests remains largely unknown [24].

Aggregation assays are influenced by GP IIb-IIIa function downstream of the outside-in signals generated by platelet agonists, including ADP, and are dependent as well on inside-out signals required for activation of the integrin. For these reasons, which may confound the interpretation of the functional state of P2Y_12_, we elected to measure surface translocation of P-selectin as an activation marker possibly more directly linked to ADP-induced platelet stimulation, as well as PAC-1 binding that, by reflecting GP IIb-IIIa activation, provides information consistent with that of aggregation assays. In order to obtain strong platelet stimulation, a desirable condition to study the effect of anti-platelet drugs, we used a combination of ADP and collagen to activate two distinct signalling pathways. Moreover, we stimulated platelets in the presence of PG E_1_, a condition occurring in the VerifyNow-P2Y_12 _test as well, with the intent of inhibiting signalling downstream of the P2Y_1 _ADP receptor and highlighting the functional state of P2Y_12_, which is the specific target of thienopyridines such as clopidogrel [25]. Of note, the use of PG E_1 _is not compatible with aggregation studies. CD62P expression and PAC-1 binding appeared equally useful as indicators of the effects of anti-platelet treatment. Because we collected blood specimens with the addition of PG E_1 _(to prevent post-sampling artefacts) we could verify that, in spite of the presence of this inhibitor, dual agonist-induced activation still caused an enhancement of activation markers, and the difference between normal controls and patients with stable coronary artery disease receiving anti-platelet drugs remained highly significant.

### Study limitations

A possible limitation of our study may be the use of the first quartile in a relatively small sample of normal population as a discriminator for low responders. Consequently, our results must be viewed only as a first step, albeit necessary, towards prospective studies (in a very large series of patients) with clinical end-points aimed at establishing therapeutic efficacy in relation to the results of laboratory tests. It is noteworthy, in this regard, that measuring the surface translocation of P-selectin in platelets stimulated in the presence of PG E_1 _appears to be more sensitive to the therapeutic inhibition of P2Y_12 _than measuring GP IIb-IIIa activation (as reflected by PAC-1 binding). In fact, several patients treated with the aspirin/clopidogrel combination had levels of P-selectin surface translocation below the 1^st ^quartile of normal values when PAC-1 binding was above this limit. Such experimental evidence is compatible with a potential lower sensitivity of aggregation assays to the effects of clopidogrel treatment, while stressing the difficulty of choosing a laboratory test for monitoring therapy without the benefit of clinical information.

Previous studies [26] have compared the VerifyNow-P2Y_12 _test (reporting only absolute PRU) with LTA, but without addressing the question of the optimum cut-off values to discriminate responders from non-responders among patients receiving anti-platelet therapy. Our results show that, in this regard, the accuracy of the VerifyNow-P2Y_12 _test is comparable to that of measuring platelet activation markers by flow cytometry, and provide a calibration of the test with the definition of more realistic cut-off values to judge the efficacy of treatment. Another limitation is that we did not evaluate possible improvements in patients with a low % inhibition following a higher dosage of clopidogrel. Our aim, nonetheless, was to validate an assay and establish an initial "reference value" in view of future prospective studies.

## Conclusion

In conclusion, these results provide information on the cut-off values, using the VerifyNow-P2Y_12 _test, that identify patients with a low response to antiplatelet therapy. A % of inhibition ≤ 15% and absolute PRU > 213 appear to reflect low platelet inhibition.

## Abbreviations

**ADP**: adenosine diphosphate; **PG**: prostaglandin; **PCI**: percutaneous coronary intervention; **PRU**: P2Y_12 _reactive units; **CD62P**: P-selectin; **mAb**: monoclonal antibody; **GP**: glycoprotein; **PRP**: platelet-rich plasma; **FITC**: fluorescein isothiocyanate; **PE**: R-phycoerythrin; **FS**: forward light scatter; **SS**: side light scatter; **CD42b**: GP Ibα; **TRAP**: thrombin receptor activating peptide; **IQR**: interquartile range; **ROC**: receiver operating characteristic; **LTA**: light transmission aggregometry.

## Competing interests

The authors do not have any financial associations to disclose that might pose a conflict of interest in connection with the submitted article. The Endotech S.PA., Italy, provided VerifyNow device and kit support for this study. The sponsor had no role in the study design, data analysis, and manuscript writing.

## Authors' contributions

Each author have contributed significantly to the submitted work, specifically: Dr CG wrote the manuscript and Drs LM, FF, MC, AC, AC were responsible for the collection analysis and interpretation of the data. Drs AC, AL, AS, JC revised the manuscript for intellectual content and Dr GC revised the manuscript for the statistical methods. Drs CG, Prof. AM, Prof. ZR and Dr. AC designed the study and gave final approval of the manuscript. All the authors have read and approved this paper.
